# Socio-Economic and Rural-Urban Differences in Healthcare and Catastrophic Health Expenditure Among Cancer Patients in China: Analysis of the China Health and Retirement Longitudinal Study

**DOI:** 10.3389/fpubh.2021.779285

**Published:** 2022-01-11

**Authors:** Yang Zhao, Shenglan Tang, Wenhui Mao, Tomi Akinyemiju

**Affiliations:** ^1^Duke Global Health Institute, Duke University, Durham, NC, United States; ^2^Global Health Research Center, Duke Kunshan University, Kunshan, China; ^3^The George Institute for Global Health at Peking University Health Science Center, Beijing, China; ^4^Department of Population Health Sciences, Duke University School of Medicine, Durham, NC, United States; ^5^Duke Cancer Institute, Duke University, Durham, NC, United States

**Keywords:** cancer study, healthcare, health expenditure, rural-urban difference, China

## Abstract

**Objective:** In China, cancer accounts for one-fifth of all deaths, and exerts a heavy toll on patients, families, healthcare systems, and society as a whole. This study aims to examine the temporal trends in socio-economic and rural-urban differences in treatment, healthcare service utilization and catastrophic health expenditure (CHE) among adult cancer patients in China. We also investigate the relationship between different types of treatment and healthcare service utilization, as well as the incidence of CHE.

**Materials and Methods:** We analyzed data from the 2011 and 2015 China Health and Retirement Longitudinal Study, a nationally representative survey including 17,224 participants (234 individuals with cancer) in 2011 and 19,569 participants (368 individuals with cancer) in 2015. The study includes six different types of cancer treatments: Chinese traditional medication (TCM); western modern medication (excluding TCM and chemotherapy medications); a combination of TCM & western medication; surgery; chemotherapy; and radiation therapy. Multivariable regression models were performed to investigate the association between cancer treatments and healthcare service utilization and CHE.

**Results:** The age-adjusted prevalence of cancer increased from 1.37% to 1.84% between 2011 and 2015. More urban patients (54%) received cancer treatment than rural patients (46%) in 2015. Patients with high socio-economic status (SES) received a higher proportion of surgical and chemotherapy treatments compared to patients with low SES in 2015. Incidence of CHE declined by 22% in urban areas but increased by 31% in rural areas. We found a positive relationship between cancer treatment and outpatient visits (OR = 2.098, 95% CI = 1.453, 3.029), hospital admission (OR = 1.961, 95% CI = 1.346, 2.857) and CHE (OR = 1.796, 95% CI = 1.231, 2.620). Chemotherapy and surgery were each associated with a 2-fold increased risk of CHE.

**Conclusions:** Significant improvements in health insurance benefit packages are necessary to ensure universal, affordable and patient-centered health coverage for cancer patients in China.

## Introduction

Non-communicable diseases are the leading cause of death and disability worldwide, and cancer is one of the most common. In China, there were an estimated 4 million new cancer cases, and 3 million cancer deaths in 2018, which accounted for a high percentage of total cancer cases (about 23%) and deaths (30%) worldwide (1). In China, cancer accounts for one-fifth of all deaths, and exerts a heavy toll on patients, families and the whole healthcare system.

To mitigate the healthcare and financial burden of non-communicable diseases including cancers, the Chinese central government launched a new round of comprehensive healthcare system reforms in 2009, focusing on healthcare delivery, essential medicines and financial protection. Most of the reform measures were designed to improve access to good quality and affordable care for patients with non-communicable diseases (2, 3). In 2015, the Urban Residents Basic Medical Insurance and New Rural Cooperative Medical System were integrated into one urban–rural resident health insurance scheme, which improved financial risk protection for cancer patients (4, 5). Additionally, the Critical Illness Health Insurance, supplementary medical insurance program, and the consolidation of medical institutions were designed to provide better benefits packages to citizens with severe chronic diseases (6).

We hypothesize that reforms in health insurance and healthcare delivery systems could further reduce catastrophic health expenditures (CHE) among cancer patients in both rural and urban regions of China. In this study, CHE is defined as the point at which annual household healthcare expenses exceed 40% of non-food household expenditure. Although a new round of health reforms have been implemented at the national level, it may take time for their impact to materialize across populations with different SES (7).

Previous research (including research in China) has shown that higher SES is associated with a greater likelihood of routine cancer screening, incidence of cancer, treatment utilization as well as better outcomes in cancer patients (8–13). Other studies in China have found a higher incidence of common cancers in men than women in most age groups (14, 15). Previous research has explored the socio-economic differences among patients with non-communicable diseases, such as hypertension, diabetes, stroke, chronic obstructive pulmonary disease, and patients with multiple chronic diseases (16–21). However, there is limited research on the financial burden caused by cancer on different socio-economic groups in China (22). Furthermore, few studies have evaluated whether CHE varies by treatment type (e.g., Chinese traditional or western medicine), or estimated the impact of the 2009 health reforms on health service utilization among cancer patients (23–26).

Our research aims include: (1) investigating temporal changes in the financial burden of treatment and CHE among Chinese adults with cancer between 2011 and 2015; (2) assessing differences in cancer treatment, health service utilization and CHE between patients of different SES and rural-urban residences; and (3) examining relationships between different types of cancer treatments and healthcare service utilization and CHE.

## Materials and Methods

### Data Source

Data were obtained from the China Health and Retirement Longitudinal Study (CHARLS) in 2011 and 2015. The CHARLS is a biennial survey, aimed to be representative of Chinese adults aged 45 years and above. The CHARLS study design is similar to the Health and Retirement Study (HRS) and other established aging-related surveys (27). Study questionnaires collect information on demographics, functional health status, healthcare and insurance, household income and expenditure, and clinical risk factors (such as blood pressure). Further details of the study methodology are published elsewhere (27).

To ensure ample representativeness at the national level, CHARLS sampled 150 counties and 450 villages/urban communities across 28 provinces, using multi-stage stratified probability-proportionate-to-size sampling. A total of 17,708 individuals were interviewed in 2011 (baseline) and 21,097 in 2015 (3rd wave). Final data were available for 17,224 participants in 2011 and 19,569 participants in 2015, after excluding participants with missing values. A total of 234 individuals (in 2011) and 368 (in 2015) self-reported having clinically-diagnosed cancer.

### Cancer Care Indicators

We identified six types of cancer treatments: Chinese traditional medication (TCM); western modern medication (taking western medication excluding TCM and chemotherapy medications for cancer treatment); a combination of TCM & western medication; surgery; chemotherapy; and radiation therapy. Overall, treatment was defined as the receipt of any TCM or western medical treatment (having one or more of the six therapies). In terms of health service utilization, this study included:(1) outpatient care (participants were asked whether they had received any outpatient care during the last month); (2) inpatient care (participants were asked whether they were hospitalized and for how many nights during the last year). Information on medical expenditures was also collected, including: total health expenditure; reimbursement; and out-of-pocket spending on outpatient services in the past month and for inpatient services in the past year.

We used CHE to measure the financial risk or economic burden on households with a family member diagnosed with cancer. There were two common criteria to meet the definition of CHE: (1) out-of-pocket expenditure (OOPE) over 40% of the household's income after paying essential living expenses (using household expenditure on non-food consumption as proxy); and (2) over 10% of total household income/expenditure (28–30). The OOPE health expenditure and household expenditure on non-food consumption were considered as the numerator and denominator, respectively.

SES was assessed using the Socio-economic Index score, and calculated based on educational attainment, occupation, and household consumption expenditure. Li's scale for Chinese residents (version 2010) (31) was used as a standard scale. This scale, commonly used in social science research in China (32–35), was modified based on a scale first proposed by Duncan (36). Education level, occupation, and household consumption expenditure were classified to assign scores, and then summarized as a comprehensive Socio-economic Index score. Based on the Socio-economic Index scores, all subjects were classified as being of either low (<10 score) or high SES (≥10 score).

### Statistical Analysis

This study applied Chi-square tests to examine the SES differences in types of cancer treatment, outpatient and inpatient service utilization, as well as incidence of CHE. For continuous variables including nights of hospitalization and OOPE, we used non-parametric tests to analyze SES group differences. Based on the pooled two-wave data of cancer patients, we performed multivariable logistic regressions to investigate the association between cancer treatment with outpatient visits, hospitalization, and incidence of CHE, adjusting for socio-demographic factors. Covariates in the regression analyses included gender, age, marital status, location of residence, region in China, and enrollment in social health insurance.

To explore differential relationships across the SES groups, we conducted subgroup analyses with logistic regression models stratified by the SES Index. The adjusted odds ratio (OR) and 95% confidence intervals (CI) were reported for the logistic regression analyses in this study. The weighted prevalence of cancers was also reported considering nonresponse data and the complex, multistage design of the CHARLS study. *P*-values less than 0.1 were considered as statistically significant. Statistical analyses were conducted using STATA software (version 15.0; StataCorp LLC College Station, Texas, United States).

## Results

Table 1 presents the socio-demographic characteristics of participants and cancer patients among individuals aged 45 years and older in 2011 and 2015. The prevalence of cancer increased from 1.36% (234 of 17,224) in 2011 to 1.88% (368 of 19,569) in 2015. The age-adjusted prevalence of cancer was 1.37% in 2011 and 1.84% in 2015. The prevalence of cancer was higher in individuals who were female, had social health insurance, were located in the eastern region, and unemployed; compared with participants who were male, did not have health insurance, lived in the western region, and were employed.

**Table 1:** The prevalence of cancer among Chinese adults in 2011 and 2015.

**Variables**	**2011**				**2015**			
	***N***	***n***	**% ^**(a)**^**	**% ^**(b)**^**	***N***	***n***	**% ^(a)^**	**% ^(b)^**
**Total**	17,224	234	1.36	1.25	19,569	368	1.88	2.05
**Gender**								
Male	8,397	86	1.02	**0.90**	9,526	116	1.22	**1.56**
Female	8,827	148	1.68	**1.58**	10,043	252	2.51	**2.51**
**Age (years)**								
45-55	6,255	89	1.42	1.23	6,699	116	1.73	2.14
55-65	6,355	76	1.20	1.16	6,611	134	2.03	2.09
≥65	4,614	69	1.50	1.38	6,259	118	1.89	1.90
**Marital status**								
Married/partnered	14,970	214	1.43	**1.32**	16,891	324	1.92	2.07
Never married/divorced	2,254	20	0.89	**0.88**	2,678	44	1.64	1.91
**Residence location**								
Urban area	6,967	103	1.48	1.18	7,908	164	2.07	2.30
Rural area	10,257	131	1.28	1.32	11,661	204	1.75	1.79
**Region**								
East	6,572	112	1.70	**1.47**	7,477	156	2.09	**2.50**
Central	6,489	78	1.20	**1.17**	7,236	137	1.89	**1.84**
West	4,163	44	1.06	**0.96**	4,856	75	1.54	**1.50**
**Health insurance**								
No	1,352	13	0.96	0.70	3,109	42	1.35	**1.23**
Yes	15,872	221	1.39	1.30	16,460	326	1.98	**2.23**
**Education level**								
Primary school/below	11,476	156	1.36	1.25	13,517	258	1.91	2.16
Middle school/above	5,748	78	1.36	1.26	6,052	110	1.82	1.84
**Employment status**								
No	5,817	113	1.94	**1.73**	6,791	193	2.84	**2.70**
Yes	11,407	121	1.06	**0.95**	12,778	175	1.37	**1.63**

*% (a), the unweighted prevalence of cancers; % (b), the weighted prevalence of cancers. The age-adjusted prevalence of cancer is 1.37% for 2011 and 1.84% for 2015*.

*Values in bold suggested a statistical significance*.

Overall, approximately half of the cancer patients utilized treatment, with a higher proportion of urban residents (54%) than rural residents (46%) receiving treatment in 2015. In addition, a higher proportion of high vs. low SES patients utilized treatment. Western medication and surgery were the two main types of treatment. People with a high SES level received more western medication treatment, surgery, and chemotherapy than low SES patients (Table 2).

**Table 2:** The proportion of cancer treatment in China, by the socioeconomic group.

**Variables**	**2011**				**2015**			
	***N***	***n***	***%***	***P-value***	***N***	***n***	***%***	***P-value***
Overall treatment*								
Urban area	103	54	52.43	0.937	164	89	54.27	**0.098**
Rural area	131	68	51.91		204	93	45.59	
SES Index, low level	156	81	51.92	0.926	191	89	46.60	0.254
SES Index, high level	78	41	52.56		177	93	52.54	
All	234	122	52.14		368	182	49.46	
**TCM only**								
Urban area	103	4	3.88	0.478	164	12	7.32	**0.094**
Rural area	131	3	2.29		204	7	3.43	
SES Index, low level	156	4	2.56	0.587	191	8	4.19	0.380
SES Index, high level	78	3	3.85		177	11	6.21	
All	234	7	2.99		368	19	5.16	
Western medication only**								
Urban area	103	28	27.18	0.372	164	30	18.29	0.776
Rural area	131	29	22.14		204	35	17.16	
SES Index, low level	156	36	23.08	0.518	191	29	15.18	0.195
SES Index, high level	78	21	26.92		177	36	20.34	
All	234	57	24.36		368	65	17.66	
**TCM & Western medication**								
Urban area	103	8	7.77	0.266	164	25	15.24	0.329
Rural area	131	16	12.21		204	24	11.76	
SES Index, low level	156	18	11.54	0.361	191	22	11.52	0.292
SES Index, high level	78	6	7.69		177	27	15.25	
All	234	24	10.26		368	49	13.32	
**Chemotherapy**								
Urban area	103	15	14.56	0.199	164	25	15.24	0.907
Rural area	131	12	9.16		204	32	15.69	
SES Index, low level	156	17	10.90	0.664	191	28	14.66	0.648
SES Index, high level	78	10	12.82		177	29	16.38	
All	234	27	11.54		368	57	15.49	
Surgery***								
Urban area	103	28	27.18	0.664	164	47	28.66	0.314
Rural area	131	39	29.77		204	49	24.02	
SES Index, low level	156	47	30.13	0.474	191	41	21.47	**0.036**
SES Index, high level	78	20	25.64		177	55	31.07	
All	234	67	28.63		368	96	26.09	
**Radiation therapy**								
Urban area	103	7	6.80	0.463	164	14	8.54	0.429
Rural area	131	6	4.58		204	13	6.37	
SES Index, low level	156	9	5.77	0.840	191	14	7.33	0.996
SES Index, high level	78	4	5.13		177	13	7.34	
All	234	13	5.56		368	27	7.34	

* *Overall treatment defined as receipt of any TCM or Western medicine treatment*.

** *Western modern medication in this study exclude chemotherapy medications*.

*** *Surgery, chemotherapy and radiation each evaluated separately, although patients might receive a combination of all three*.

*P-values in bold are statistically significant*.

There was increasing health service utilization among cancer patients in China from 2011 to 2015 (outpatient visit, 26 to 30%; admission rate, 23 to 30%; average nights of hospitalization, 3.41 to 3.81). In 2011, residents living in rural areas had a higher proportion of outpatient visits but less OOPE for outpatient care than urban residents. However, by 2015, this gap had narrowed and the OOPE for outpatient care had more than tripled in rural areas, and declined significantly in urban areas. Between 2011 and 2015, CHE declined by 22% in urban areas (25% in 2011 and 19% in 2015) but increased by 31% in rural areas (25% in 2011 to 33% in 2015). In 2011, low SES cancer patients had significantly lower OOPE for inpatient care compared with high SES patients. However, by 2015 this gap had narrowed and was no longer significant (Table 3).

**Table 3:** Health service utilization and health spending among cancer patients in China, by the socioeconomic group.

**Variables**	**2011**		**2015**	
	**%**	***P*** **-value**	**%**	***P*** **-value**
**Outpatient visits, last month (%)**				
Urban area	18.45	**0.007**	26.22	0.115
Rural area	34.35		33.82	
SES Index, low level	23.72	0.247	29.84	0.798
SES Index, high level	30.77		31.07	
All	26.07		30.43	
**Admission rate, last year (%)**				
Urban area	25.24	0.401	32.32	0.362
Rural area	20.61		27.94	
SES Index, low level	21.15	0.439	25.65	**0.065**
SES Index, high level	25.64		34.46	
All	22.65		29.89	
**Nights in hospital, last year (median; mean)**				
Urban area	0; 4.20	0.252	0; 4.09	0.230
Rural area	0; 2.79		0; 3.58	
SES Index, low level	0; 2.96	0.211	0; 3.46	0.126
SES Index, high level	0; 4.32		0; 4.18	
All	0; 3.41		0; 3.81	
OOPE for outpatient care*, CNY (median; mean)				
Urban area	500; 4,494	**0.082**	300; 1,564	0.410
Rural area	100; 517		500; 3,893	
SES Index, low level	200; 2,121	**0.005**	300; 2,225	0.214
SES Index, high level	160; 1,093		500; 3,826	
All	190; 1,820		400; 3,025	
OOPE for inpatient care**, CNY (median; mean)				
Urban area	7,500; 19,612	**0.087**	4,500; 16,566	0.967
Rural area	3,000; 12,477		4,000; 16,153	
SES Index, low level	3,000; 10,372	**0.022**	4,000; 13,851	0.584
SES Index, high level	10,000; 25,225		5,000; 18,360	
All	6,000; 15,977		4,100; 16,352	
**Catastrophic health expenditure (%)**				
Urban area	25.24	0.993	19.51	**0.003**
Rural area	25.19		33.33	
SES Index, low level	26.28	0.595	28.80	0.468
SES Index, high level	23.08		25.42	
All	25.21		27.17	

*In this study, we defined catastrophic health expenditure as medical OOPE equalling or exceeding 40% of the household's expenditure on non-food consumption. CNY, Chinese Yuan*.

*P-values in bold are statistically significant*.

* *Out-of-pocket expenditure among cancer patients with outpatient visit*.

** *Out-of-pocket expenditure among cancer patients with inpatient care*.

The multivariable regression analyses suggest positive relationships between cancer treatment and outpatient visits (OR = 2.098, 95% CI =1.453, 3.029), admission to hospital (OR = 1.961, 95% CI = 1.346, 2.857) and CHE (OR = 1.796, 95% CI =1.231, 2.620). Chemotherapy (OR = 2.53, 95% CI: 1.55, 4.12) and surgery (surgery: OR = 2.15, 95% CI: 1.44, 3.20) were each associated with a 2-fold increased risk of CHE, after controlling for all socio-demographic covariates. This association was stronger among high SES groups (chemotherapy OR = 3.16, 95% CI: 1.44, 6.90; surgery: OR = 2.36, 95% CI: 1.24, 4.49) compared with low SES groups (chemotherapy OR = 2.77, 95% CI: 1.41, 5.41; surgery OR = 2.07, 95% CI: 1.22, 3.51). There were no significant associations observed for TCM with CHE overall or by SES (Table 4).

**Table 4:** Differential impacts of the cancer treatment on health service use and catastrophic health expenditure.

**Treatment type**	**Outpatient visits**		**Admission to hospital**		**Catastrophic health expenditure**	
	**OR**	***P*** **-value**	**OR**	***P*** **-value**	**OR**	***P*** **-value**
**All cancer patients**						
Overall cancer treatment*	2.098	**<0.001**	1.961	**<0.001**	1.796	**0.002**
TCM only	2.002	**0.095**	0.771	0.593	1.187	0.709
Western medication only**	1.227	0.360	1.503	**0.068**	1.204	0.420
TCM & western medication	1.904	**0.014**	1.001	0.996	1.201	0.511
Chemotherapy	1.823	**0.017**	3.622	**<0.001**	2.530	**<0.001**
Surgery***	1.750	**0.005**	2.041	**<0.001**	2.146	**<0.001**
Radiation therapy	0.845	0.660	3.310	**<0.001**	1.675	0.138
**SES Index, low level**						
Overall cancer treatment	1.637	**0.044**	1.690	**0.047**	1.416	0.157
TCM only	2.740	0.100	0.662	0.605	1.367	0.622
Western medication only	1.348	0.333	1.027	0.936	0.819	0.538
TCM & western medication	1.570	0.208	0.997	0.994	0.948	0.888
Chemotherapy	2.222	**0.023**	3.496	**<0.001**	2.766	**0.003**
Surgery	1.422	0.197	1.811	**0.039**	2.072	**0.007**
Radiation therapy	1.314	0.566	3.827	**0.003**	1.663	0.265
**SES Index, high level**						
Overall cancer treatment	3.017	**<0.001**	2.373	**0.003**	2.651	**0.003**
TCM only	1.321	0.644	0.791	0.708	0.956	0.949
Western medication only	1.210	0.572	2.325	**0.008**	1.791	**0.091**
TCM & western medication	2.087	**0.065**	0.916	0.830	1.629	0.247
Chemotherapy	1.338	0.443	3.864	**<0.001**	3.156	**0.004**
Surgery	2.223	**0.008**	2.404	**0.003**	2.364	**0.009**
Radiation therapy	0.228	**0.062**	2.457	**0.089**	1.940	0.245

*Logistic regressions adjusted for: age, gender, marital status, residence location, region, health insurance. Values in bold suggested a statistical significance*.

* *Overall treatment defined as receipt of any TCM or Western medicine treatment*.

** *Western modern medication in this study exclude chemotherapy medications*.

*** *Surgery, chemotherapy and radiation each evaluated separately, although patients might receive a combination of all three*.

## Discussion

As this nationally representative study indicated, there were increasing trends in the prevalence of cancer, and healthcare service utilization among adults with cancer from 2011 to 2015. The results suggest socio-economic and rural-urban differences impact cancer treatment in China. For example, about half of cancer patients in urban areas utilized cancer treatment in 2015, a higher proportion than rural residents. Patients with high SES received a higher proportion of surgery and chemotherapy treatment compared to those with low SES in 2015. Moreover, there was a substantial increase in CHE among rural patients between 2011 and 2015, but a substantial decrease among urban patients in during the same time period. Regression analyses revealed that the cost of chemotherapy and surgery appeared to drive the CHE increase, regardless of SES.

There are likely several factors contributing to the observed urban-rural differences in cancer treatment that need to be addressed in order to improve equitable access to healthcare. First, cancer treatment is a specialized service, and generally only secondary or higher levels hospitals have the capacity to provide it. Therefore, rural residents have less geographic access to treatment compared with urban areas. Additionally, some rural patients require extended travel and incur additional expenses to receive treatment in urban areas (37).

Second, previous studies have documented the disparities in access to healthcare between urban and rural areas in China (38, 39). Rural areas are less likely to have access to the same quality of healthcare services as urban areas due to differences in economic development (38, 40). Rural areas are more likely to have a shortage of healthcare service providers and lack of social support services (41–43). Previous research found that the number of licensed doctors and nurses, medical-technical personnel, and beds per 1,000 population increased more in Chinese urban areas than in rural areas from 2005 to 2017 (44). There were 2.57 more registered doctors per thousand people in urban areas than in rural areas in China in 2015 (45). As a result cancer patients in rural areas may prefer to use services in nearby urban areas despite the higher financial burden.

Third, patients in rural areas potentially face more financial barriers in accessing cancer treatment compared with patients in urban areas. This is likely due to the fact that rural areas have more barriers in physical access to healthcare services, and high per-capita payment for cancer treatment. Social medical insurance also likely contributes to the urban-rural disparity in CHE due to gaps in coverage and benefit packages (46). While over 95% of the Chinese population are covered by basic medical insurance, the benefits packages vary significantly (5, 6, 13). For example, in 2015 the per-capita fund for Urban Employee Basic Medical Insurance was US$424.7, whereas it was only $61.2 for New Rural Cooperative Medical System (15). The co-payment rate for the New Rural Cooperative Medical System (73.4%) was higher than the Urban Employee Basic Medical Insurance (36.8%) and Urban Residents Basic Medical Insurance (50.7%) in 2008 (47). Cancer patients in rural regions receive lower reimbursement rates and have lower annual maximum payments from insurance. Additionally, patients that seek treatment outside of their residential location usually receive lower reimbursement rates. For example, if cancer patients from rural areas utilize cancer treatment from another city, they have to pay a higher proportion of their bill out-of-pocket. Such a heavy financial burden likely prohibits low SES and rural patients seeking cancer treatment, possibly explaining the large urban-rural disparity in inpatient care compared to outpatient visits. More attention should be given to the financial implications caused by out of pocket expenses for cancer treatment across the different health insurance schemes in future.

Finally, while the expenditure for cancer treatment is increasing in both rural and urban areas, unequal economic development and the low amount of disposable income available to rural residents may further contribute to the urban-rural disparities. In 2015, the disposable income of urban citizens was 31,195 CNY per capita, three times higher than that of residents living in rural areas (11,422 CNY) (48). Medical expenditure due to cancer treatment, specifically surgery and chemotherapy, is likely to have significantly greater impact on rural patients, leading to increased risk of CHE and impoverishment (48). In addition, since the New Rural Cooperative Medical System and the Urban Residents Basic Medical Insurance were mainly financed by local county-level governments at the early stage of China's health system reform, the quality of benefit packages likely depends on the strength of the local economy (45).

We observed that cancer patients with high SES were less likely to experience CHE than those with low SES, and by 2015, rural cancer patients had almost double the prevalence of CHE compared with urban cancer patients, suggesting that a potential unintended consequence of the health reform is the widening of rural-urban disparities in CHE. The findings are consistent with previous research on financial burden among residents with non-communicable diseases in China (15, 49). Recent studies have documented a rapid rise in healthcare costs for cancer patients in China. However, data on the population-level economic burden of cancer is limited and the reported expenditure per patient may be underestimated (50–56). For example, a systematic review of the economic burden of liver cancer shows an increase in expenditure indicators (direct medical expenditure, annual expenditure per visit and annual expenditure per diem) from 1996 to 2015, with medication costs accounting for more than half of overall expenditure (56. 6%) (53). For colorectal cancer, the annual growth rate for medical expenditure per patient, per visit and per day increased from 6.9 to 7.8% from 1996 to 2015, respectively (54).

### Policy Implications

The health insurance programs in China have had some positive impact on healthcare utilization. For instance, we observed that outpatient visits and admissions increased between 2011 and 2015 in all socio-demographic groups. However, challenges remain. Overall, the burden of cancer among adults in China is increasing, and about one-fourth of cancer patients experienced CHE. Yet disparities among urban-rural areas, and across different SES still exist, even after the implementation of the national health insurance scheme.

To reduce financial burden of cancer and bridge the SES gap, comprehensive changes to health insurance benefit packages and healthcare resource allocation are needed to ensure universal, affordable and patient-centered health coverage. First, the Urban Residents Basic Medical Insurance and the New Rural Cooperative Medical System need to be further integrated to provide similar contributions, benefit packages, as well as financial risk protection to accelerate the equitable access to health services in both urban and rural areas. Secondly, social health insurance benefit packages need to be expanded. Health services that have proven cost-effective (including medicines) should be added to the National Insurance Reimbursable List. For instance, 17 and 22 anti-cancer medications were added to the National Insurance Reimbursable List in 2018 and 2019 respectively. This allowed for a significant price cut and helped to reduce the financial burden on cancer patients. (57). Thirdly, while the National Health Insurance provides financial protection for essential care, the Critical Illness Medical Insurance should play an increasing role in providing financial support for catastrophic expenses, including cancer treatment. In particular, the current Critical Illness Medical Insurance in most regions followed the National Insurance Reimbursable List which prioritizes essential care (58). To provide better protection against catastrophic expenses, Critical Illness Medical Insurance should explore additional coverage on other treatments with proven health benefits. Furthermore, enhancing the capacity of the National Public Health Initiative would increase cancer prevention strategies such as routine screening and case management. This might lead to early detection, reduced financial burden and improved cancer outcomes. This approach is especially critical given the healthcare disruptions caused by the COVID-19 pandemic.

### Strengths and Limitations

This research utilized data from a nationally representative study to investigate the trends and disparities in cancer treatment, healthcare service utilization and CHE from 2011 to 2015. Our study contributes to a deeper understanding of the socio-economic and rural-urban disparities in cancer treatment, health service utilization and expenditure. There are several significant limitations. This study used self-reported data on cancer diagnosis, treatment type and healthcare service utilization. Self-reported information could result in underestimated figures as a result of recall bias (53). Medical information regarding cancer severity was not collected. Using the indicator of CHE to measure the financial burden might ignore a part of patients not seeking health care because of economic restraints who could be even more vulnerable, while the CHARLS data showed that only a very small proportion of patients with cancer in China did not seek medical treatment due to economic restraints. The CHARLS survey only included middle-aged and elderly members of the population. Future research should also focus on younger adults. Moreover, about 20% of participants in the CHARLS survey had missing values for some key variables.

In conclusion, the burden of cancer among Chinese adults is increasing. Socio-economic and urban-rural disparities in cancer treatment and health service utilization were largely determined by patient financial capability. The current social health insurance schemes are insufficient to address these disparities. A comprehensive health insurance policy with expanded benefit packages and a stronger Public Medical Assistance System, are essential to providing adequate and equitable cancer treatment in China.

## Data Availability Statement

The datasets generated and analyzed during the current study are available in the China Health and Retirement Longitudinal Study repository; http://charls.pku.edu.cn/pages/data/111/en.html.

## Ethics Statement

The studies involving human participants were reviewed and approved by the Biomedical Ethics Review Committee of Peking University approved the CHARLS study, and all interviewees were required to provide informed consent. The ethical approval number was IRB00001052–11015. The patients/participants provided their written informed consent to participate in this study.

## Author Contributions

YZ and ST conceived and designed the study. YZ carried out the initial analysis. YZ, ST, and TA interpreted the data. WM and YZ analyzed the literature. YZ and WM wrote the first draft of the paper. TA, ST, and WM provided guidance on the first draft and provided feedback on intellectual content. All authors reviewed and had final approval of the submitted and published versions.

## Conflict of Interest

The authors declare that the research was conducted in the absence of any commercial or financial relationships that could be construed as a potential conflict of interest.

## Publisher's Note

All claims expressed in this article are solely those of the authors and do not necessarily represent those of their affiliated organizations, or those of the publisher, the editors and the reviewers. Any product that may be evaluated in this article, or claim that may be made by its manufacturer, is not guaranteed or endorsed by the publisher.
