# Supplementary material for: Socio-Economic and Rural-Urban Differences in Healthcare and Catastrophic Health Expenditure Among Cancer Patients in China: Analysis of the China Health and Retirement Longitudinal Study
Source: Front Public Health. 2022 Jan 11;9:779285. doi: 10.3389/fpubh.2021.779285 (PMC8787105; doi:10.3389/fpubh.2021.779285)
Supplement: Supplementary file 1 [file Table_1.docx]

**Appendix tables**

**Table S1** Differential impacts of the cancer treatment on health service use and catastrophic health expenditure

| **Treatment type** | **Outpatient**  **visits** | | | **Admission**  **to hospital** | | | **Catastrophic health expenditure** | | |
| --- | --- | --- | --- | --- | --- | --- | --- | --- | --- |
|  | OR | 95% CI | | OR | 95% CI | | OR | 95% CI | |
| **All cancer patients** |  |  |  |  |  |  |  |  |  |
| Overall cancer treatment | **2.098** | 1.453 | 3.029 | **1.961** | 1.346 | 2.857 | **1.796** | 1.231 | 2.620 |
| TCM only | 2.002 | 0.886 | 4.525 | 0.771 | 0.297 | 1.999 | 1.187 | 0.481 | 2.928 |
| Western medication only | 1.227 | 0.792 | 1.902 | 1.503 | 0.970 | 2.329 | 1.204 | 0.767 | 1.889 |
| TCM & western medication | **1.904** | 1.142 | 3.176 | 1.001 | 0.576 | 1.739 | 1.201 | 0.696 | 2.072 |
| Chemotherapy | **1.823** | 1.115 | 2.979 | **3.622** | 2.233 | 5.874 | **2.530** | 1.551 | 4.126 |
| Surgery | **1.750** | 1.187 | 2.580 | **2.041** | 1.371 | 3.037 | **2.146** | 1.437 | 3.204 |
| Radiation therapy | 0.845 | 0.400 | 1.788 | **3.310** | 1.714 | 6.393 | 1.675 | 0.847 | 3.314 |
| **SES Index, low level** |  |  |  |  |  |  |  |  |  |
| Overall cancer treatment | **1.637** | 1.013 | 2.647 | **1.690** | 1.008 | 2.833 | 1.416 | 0.875 | 2.291 |
| TCM only | 2.740 | 0.824 | 9.109 | 0.662 | 0.139 | 3.155 | 1.367 | 0.394 | 4.741 |
| Western medication only | 1.348 | 0.736 | 2.467 | 1.027 | 0.530 | 1.989 | 0.819 | 0.435 | 1.545 |
| TCM & western medication | 1.570 | 0.778 | 3.169 | 0.997 | 0.452 | 2.199 | 0.948 | 0.446 | 2.012 |
| Chemotherapy | **2.222** | 1.118 | 4.414 | **3.496** | 1.773 | 6.890 | **2.766** | 1.414 | 5.410 |
| Surgery | 1.422 | 0.833 | 2.425 | **1.811** | 1.031 | 3.183 | **2.072** | 1.224 | 3.507 |
| Radiation therapy | 1.314 | 0.518 | 3.331 | **3.827** | 1.584 | 9.246 | 1.663 | 0.680 | 4.065 |
| **SES Index, high level** |  |  |  |  |  |  |  |  |  |
| Overall cancer treatment | **3.017** | 1.645 | 5.535 | **2.373** | 1.344 | 4.190 | **2.651** | 1.390 | 5.059 |
| TCM only | 1.321 | 0.406 | 4.300 | 0.791 | 0.232 | 2.698 | 0.956 | 0.246 | 3.717 |
| Western medication only | 1.210 | 0.625 | 2.344 | **2.325** | 1.247 | 4.334 | 1.791 | 0.912 | 3.519 |
| TCM & western medication | 2.087 | 0.955 | 4.562 | 0.916 | 0.414 | 2.031 | 1.629 | 0.713 | 3.720 |
| Chemotherapy | 1.338 | 0.636 | 2.817 | **3.864** | 1.875 | 7.962 | **3.156** | 1.442 | 6.907 |
| Surgery | **2.223** | 1.232 | 4.012 | **2.404** | 1.341 | 4.311 | **2.364** | 1.243 | 4.496 |
| Radiation therapy | 0.228 | 0.048 | 1.077 | 2.457 | 0.871 | 6.931 | 1.940 | 0.634 | 5.937 |

Note: Logistic regressions adjusted for: age, gender, marital status, residence location, region, health insurance. Values in bold suggested a statistical significance (P<0.05).

*Overall treatment defined as receipt of any TCM or Western medicine treatment.

**Western modern medication in this study exclude chemotherapy medications.

***Surgery, chemotherapy and radiation each evaluated separately, although patients might receive a combination of all three.

**Table S2** The impacts of the overall cancer treatment on health service use and catastrophic health expenditure

| **Variables (reference)** | **Outpatient**  **visits** | | | | **Admission**  **to hospital** | | | | **Catastrophic health expenditure** | | | |
| --- | --- | --- | --- | --- | --- | --- | --- | --- | --- | --- | --- | --- |
|  | OR | P value | 95% CI | | OR | P value | 95% CI | | OR | P value | 95% CI | |
| Overall cancer treatment (no) |  |  |  |  |  |  |  |  |  |  |  |  |
| Yes | 2.098 | <0.001 | 1.453 | 3.029 | 1.961 | <0.001 | 1.346 | 2.857 | 1.796 | 0.002 | 1.231 | 2.620 |
| Age (45-55 years) |  |  |  |  |  |  |  |  |  |  |  |  |
| 55-65 years | 0.800 | 0.329 | 0.510 | 1.253 | 1.388 | 0.169 | 0.870 | 2.215 | 1.270 | 0.310 | 0.800 | 2.015 |
| ≥65 years | 1.069 | 0.788 | 0.658 | 1.736 | 1.448 | 0.157 | 0.867 | 2.420 | 1.326 | 0.280 | 0.795 | 2.213 |
| Gender (male) |  |  |  |  |  |  |  |  |  |  |  |  |
| Female | 1.281 | 0.227 | 0.857 | 1.914 | 0.720 | 0.102 | 0.486 | 1.068 | 0.821 | 0.332 | 0.550 | 1.224 |
| Marital status (married/partnered) |  |  |  |  |  |  |  |  |  |  |  |  |
| Never married/divorced | 1.011 | 0.972 | 0.547 | 1.868 | 0.759 | 0.413 | 0.392 | 1.470 | 0.470 | 0.042 | 0.227 | 0.975 |
| Residence location (urban area) |  |  |  |  |  |  |  |  |  |  |  |  |
| Rural area | 1.748 | 0.004 | 1.189 | 2.569 | 0.941 | 0.757 | 0.640 | 1.384 | 1.651 | 0.013 | 1.111 | 2.454 |
| Region (east) |  |  |  |  |  |  |  |  |  |  |  |  |
| Central | 0.868 | 0.502 | 0.575 | 1.311 | 1.537 | 0.045 | 1.011 | 2.338 | 1.470 | 0.074 | 0.963 | 2.243 |
| West | 0.904 | 0.687 | 0.555 | 1.474 | 1.295 | 0.322 | 0.777 | 2.158 | 1.369 | 0.226 | 0.823 | 2.278 |
| Health insurance (no) |  |  |  |  |  |  |  |  |  |  |  |  |
| Yes | 1.127 | 0.720 | 0.587 | 2.164 | 1.236 | 0.535 | 0.633 | 2.416 | 1.045 | 0.898 | 0.534 | 2.044 |
| SES Index (low level) |  |  |  |  |  |  |  |  |  |  |  |  |
| High level | 1.070 | 0.725 | 0.733 | 1.561 | 1.553 | 0.025 | 1.057 | 2.280 | 0.925 | 0.694 | 0.625 | 1.367 |
| Constant | 0.166 | <0.001 | 0.071 | 0.388 | 0.146 | <0.001 | 0.061 | 0.347 | 0.160 | <0.001 | 0.067 | 0.382 |

Note: Logistic regressions adjusted for: age, gender, marital status, residence location, region, health insurance and socioeconomic level. *Overall treatment defined as receipt of any Chinese traditional medication or western medicine treatment.

**Table S3** The impacts of the Chinese Traditional Medication only on health service use and catastrophic health expenditure

| **Variables (reference)** | **Outpatient**  **visits** | | | | **Admission**  **to hospital** | | | | **Catastrophic health expenditure** | | | |
| --- | --- | --- | --- | --- | --- | --- | --- | --- | --- | --- | --- | --- |
|  | OR | P value | 95% CI | | OR | P value | 95% CI | | OR | P value | 95% CI | |
| Chinese traditional medication (no) |  |  |  |  |  |  |  |  |  |  |  |  |
| Yes | 2.002 | 0.095 | 0.886 | 4.525 | 0.771 | 0.593 | 0.297 | 1.999 | 1.187 | 0.709 | 0.481 | 2.928 |
| Age (45-55 years) |  |  |  |  |  |  |  |  |  |  |  |  |
| 55-65 years | 0.813 | 0.361 | 0.521 | 1.268 | 1.392 | 0.161 | 0.876 | 2.210 | 1.270 | 0.307 | 0.803 | 2.009 |
| ≥65 years | 1.058 | 0.817 | 0.655 | 1.710 | 1.412 | 0.183 | 0.850 | 2.346 | 1.301 | 0.310 | 0.783 | 2.162 |
| Gender (male) |  |  |  |  |  |  |  |  |  |  |  |  |
| Female | 1.197 | 0.373 | 0.806 | 1.777 | 0.680 | 0.051 | 0.461 | 1.002 | 0.782 | 0.222 | 0.526 | 1.161 |
| Marital status (married/partnered) |  |  |  |  |  |  |  |  |  |  |  |  |
| Never married/divorced | 1.031 | 0.922 | 0.562 | 1.892 | 0.787 | 0.471 | 0.410 | 1.509 | 0.493 | 0.054 | 0.240 | 1.013 |
| Residence location (urban area) |  |  |  |  |  |  |  |  |  |  |  |  |
| Rural area | 1.713 | 0.006 | 1.170 | 2.508 | 0.909 | 0.623 | 0.620 | 1.331 | 1.609 | 0.018 | 1.086 | 2.385 |
| Region (east) |  |  |  |  |  |  |  |  |  |  |  |  |
| Central | 0.871 | 0.505 | 0.580 | 1.308 | 1.496 | 0.057 | 0.988 | 2.264 | 1.450 | 0.082 | 0.954 | 2.204 |
| West | 0.892 | 0.643 | 0.550 | 1.447 | 1.235 | 0.413 | 0.745 | 2.049 | 1.323 | 0.278 | 0.798 | 2.192 |
| Health insurance (no) |  |  |  |  |  |  |  |  |  |  |  |  |
| Yes | 1.023 | 0.944 | 0.536 | 1.955 | 1.125 | 0.727 | 0.580 | 2.185 | 0.963 | 0.911 | 0.495 | 1.872 |
| SES Index (low level) |  |  |  |  |  |  |  |  |  |  |  |  |
| High level | 1.069 | 0.725 | 0.736 | 1.553 | 1.568 | 0.020 | 1.073 | 2.291 | 0.935 | 0.733 | 0.634 | 1.377 |
| Constant | 0.277 | 0.001 | 0.126 | 0.608 | 0.250 | 0.001 | 0.112 | 0.559 | 0.248 | 0.001 | 0.111 | 0.558 |

Note: Logistic regressions adjusted for: age, gender, marital status, residence location, region, health insurance and socioeconomic level.

**Table S4** The impacts of the western medication only on health service use and catastrophic health expenditure

| **Variables (reference)** | **Outpatient**  **visits** | | | | **Admission**  **to hospital** | | | | **Catastrophic health expenditure** | | | |
| --- | --- | --- | --- | --- | --- | --- | --- | --- | --- | --- | --- | --- |
|  | OR | P value | 95% CI | | OR | P value | 95% CI | | OR | P value | 95% CI | |
| Western medication only (no) |  |  |  |  |  |  |  |  |  |  |  |  |
| Yes | 1.227 | 0.360 | 0.792 | 1.902 | 1.503 | 0.068 | 0.970 | 2.329 | 1.204 | 0.420 | 0.767 | 1.889 |
| Age (45-55 years) |  |  |  |  |  |  |  |  |  |  |  |  |
| 55-65 years | 0.797 | 0.315 | 0.511 | 1.241 | 1.382 | 0.172 | 0.869 | 2.198 | 1.257 | 0.329 | 0.795 | 1.987 |
| ≥65 years | 1.032 | 0.898 | 0.640 | 1.664 | 1.395 | 0.200 | 0.839 | 2.320 | 1.284 | 0.335 | 0.773 | 2.132 |
| Gender (male) |  |  |  |  |  |  |  |  |  |  |  |  |
| Female | 1.195 | 0.376 | 0.805 | 1.775 | 0.691 | 0.063 | 0.468 | 1.021 | 0.786 | 0.232 | 0.529 | 1.167 |
| Marital status (married/partnered) |  |  |  |  |  |  |  |  |  |  |  |  |
| Never married/divorced | 1.052 | 0.870 | 0.573 | 1.931 | 0.824 | 0.561 | 0.428 | 1.584 | 0.502 | 0.061 | 0.244 | 1.034 |
| Residence location (urban area) |  |  |  |  |  |  |  |  |  |  |  |  |
| Rural area | 1.676 | 0.007 | 1.148 | 2.446 | 0.916 | 0.652 | 0.626 | 1.342 | 1.603 | 0.018 | 1.083 | 2.372 |
| Region (east) |  |  |  |  |  |  |  |  |  |  |  |  |
| Central | 0.866 | 0.488 | 0.577 | 1.300 | 1.519 | 0.049 | 1.002 | 2.301 | 1.451 | 0.082 | 0.954 | 2.207 |
| West | 0.873 | 0.581 | 0.539 | 1.413 | 1.227 | 0.428 | 0.739 | 2.037 | 1.310 | 0.294 | 0.791 | 2.170 |
| Health insurance (no) |  |  |  |  |  |  |  |  |  |  |  |  |
| Yes | 1.051 | 0.881 | 0.549 | 2.010 | 1.188 | 0.613 | 0.610 | 2.314 | 0.987 | 0.969 | 0.506 | 1.925 |
| SES Index (low level) |  |  |  |  |  |  |  |  |  |  |  |  |
| High level | 1.070 | 0.722 | 0.737 | 1.552 | 1.546 | 0.025 | 1.057 | 2.262 | 0.930 | 0.714 | 0.631 | 1.371 |
| Constant | 0.277 | 0.002 | 0.125 | 0.613 | 0.213 | <0.001 | 0.094 | 0.482 | 0.238 | 0.001 | 0.105 | 0.538 |

Note: Logistic regressions adjusted for: age, gender, marital status, residence location, region, health insurance and socioeconomic level. *Western medication in this study exclude chemotherapy medications.

**Table S5** The impacts of the TCM & Western medication on health service use and catastrophic health expenditure

| **Variables (reference)** | **Outpatient**  **visits** | | | | **Admission**  **to hospital** | | | | **Catastrophic health expenditure** | | | |
| --- | --- | --- | --- | --- | --- | --- | --- | --- | --- | --- | --- | --- |
|  | OR | P value | 95% CI | | OR | P value | 95% CI | | OR | P value | 95% CI | |
| TCM & Western medication (no) |  |  |  |  |  |  |  |  |  |  |  |  |
| Yes | 1.904 | 0.014 | 1.142 | 3.176 | 1.001 | 0.996 | 0.576 | 1.739 | 1.201 | 0.511 | 0.696 | 2.072 |
| Age (45-55 years) |  |  |  |  |  |  |  |  |  |  |  |  |
| 55-65 years | 0.784 | 0.285 | 0.503 | 1.224 | 1.398 | 0.156 | 0.880 | 2.220 | 1.261 | 0.321 | 0.798 | 1.993 |
| ≥65 years | 1.043 | 0.863 | 0.646 | 1.685 | 1.419 | 0.176 | 0.854 | 2.358 | 1.298 | 0.314 | 0.781 | 2.156 |
| Gender (male) |  |  |  |  |  |  |  |  |  |  |  |  |
| Female | 1.208 | 0.350 | 0.813 | 1.796 | 0.682 | 0.053 | 0.462 | 1.005 | 0.785 | 0.229 | 0.528 | 1.165 |
| Marital status (married/partnered) |  |  |  |  |  |  |  |  |  |  |  |  |
| Never married/divorced | 1.012 | 0.970 | 0.550 | 1.863 | 0.788 | 0.473 | 0.411 | 1.511 | 0.490 | 0.053 | 0.238 | 1.008 |
| Residence location (urban area) |  |  |  |  |  |  |  |  |  |  |  |  |
| Rural area | 1.695 | 0.007 | 1.157 | 2.481 | 0.916 | 0.651 | 0.626 | 1.341 | 1.605 | 0.018 | 1.084 | 2.377 |
| Region (east) |  |  |  |  |  |  |  |  |  |  |  |  |
| Central | 0.842 | 0.410 | 0.560 | 1.267 | 1.503 | 0.054 | 0.993 | 2.275 | 1.437 | 0.090 | 0.945 | 2.186 |
| West | 0.831 | 0.455 | 0.510 | 1.352 | 1.247 | 0.393 | 0.752 | 2.068 | 1.300 | 0.309 | 0.784 | 2.156 |
| Health insurance (no) |  |  |  |  |  |  |  |  |  |  |  |  |
| Yes | 0.984 | 0.961 | 0.516 | 1.877 | 1.128 | 0.723 | 0.581 | 2.191 | 0.951 | 0.883 | 0.489 | 1.849 |
| SES Index (low level) |  |  |  |  |  |  |  |  |  |  |  |  |
| High level | 1.066 | 0.737 | 0.733 | 1.550 | 1.563 | 0.021 | 1.069 | 2.284 | 0.934 | 0.730 | 0.634 | 1.376 |
| Constant | 0.285 | 0.002 | 0.130 | 0.623 | 0.244 | 0.001 | 0.109 | 0.544 | 0.250 | 0.001 | 0.112 | 0.558 |

Note: Logistic regressions adjusted for: age, gender, marital status, residence location, region, health insurance and socioeconomic level. TCM refers to traditional Chinese medication.*Western medication in this study exclude chemotherapy medications.

**Table S6** The impacts of the Chemotherapy on health service use and catastrophic health expenditure

| **Variables (reference)** | **Outpatient**  **visits** | | | | **Admission**  **to hospital** | | | | **Catastrophic health expenditure** | | | |
| --- | --- | --- | --- | --- | --- | --- | --- | --- | --- | --- | --- | --- |
|  | OR | P value | 95% CI | | OR | P value | 95% CI | | OR | P value | 95% CI | |
| Chemotherapy (no) |  |  |  |  |  |  |  |  |  |  |  |  |
| Yes | 1.823 | 0.017 | 1.115 | 2.979 | 3.622 | <0.001 | 2.233 | 5.874 | 2.530 | <0.001 | 1.551 | 4.126 |
| Age (45-55 years) |  |  |  |  |  |  |  |  |  |  |  |  |
| 55-65 years | 0.779 | 0.273 | 0.499 | 1.217 | 1.342 | 0.225 | 0.835 | 2.158 | 1.226 | 0.389 | 0.771 | 1.950 |
| ≥65 years | 1.037 | 0.880 | 0.642 | 1.676 | 1.447 | 0.164 | 0.860 | 2.435 | 1.312 | 0.300 | 0.785 | 2.192 |
| Gender (male) |  |  |  |  |  |  |  |  |  |  |  |  |
| Female | 1.253 | 0.268 | 0.841 | 1.868 | 0.748 | 0.153 | 0.502 | 1.114 | 0.844 | 0.410 | 0.565 | 1.262 |
| Marital status (married/partnered) |  |  |  |  |  |  |  |  |  |  |  |  |
| Never married/divorced | 1.049 | 0.877 | 0.571 | 1.927 | 0.809 | 0.535 | 0.414 | 1.581 | 0.495 | 0.058 | 0.239 | 1.025 |
| Residence location (urban area) |  |  |  |  |  |  |  |  |  |  |  |  |
| Rural area | 1.689 | 0.007 | 1.154 | 2.471 | 0.926 | 0.699 | 0.626 | 1.369 | 1.641 | 0.015 | 1.102 | 2.442 |
| Region (east) |  |  |  |  |  |  |  |  |  |  |  |  |
| Central | 0.855 | 0.452 | 0.569 | 1.286 | 1.541 | 0.047 | 1.006 | 2.359 | 1.457 | 0.082 | 0.953 | 2.227 |
| West | 0.913 | 0.714 | 0.563 | 1.483 | 1.406 | 0.198 | 0.837 | 2.362 | 1.422 | 0.178 | 0.852 | 2.372 |
| Health insurance (no) |  |  |  |  |  |  |  |  |  |  |  |  |
| Yes | 1.023 | 0.946 | 0.535 | 1.954 | 1.142 | 0.702 | 0.578 | 2.258 | 0.962 | 0.909 | 0.492 | 1.881 |
| SES Index (low level) |  |  |  |  |  |  |  |  |  |  |  |  |
| High level | 1.060 | 0.760 | 0.729 | 1.541 | 1.563 | 0.025 | 1.059 | 2.307 | 0.923 | 0.689 | 0.624 | 1.366 |
| Constant | 0.262 | 0.001 | 0.119 | 0.576 | 0.176 | <0.001 | 0.077 | 0.405 | 0.202 | <0.001 | 0.089 | 0.459 |

Note: Logistic regressions adjusted for: age, gender, marital status, residence location, region, health insurance and socioeconomic level.

**Table S7** The impacts of the Surgery on health service use and catastrophic health expenditure

| **Variables (reference)** | **Outpatient**  **visits** | | | | **Admission**  **to hospital** | | | | **Catastrophic health expenditure** | | | |
| --- | --- | --- | --- | --- | --- | --- | --- | --- | --- | --- | --- | --- |
|  | OR | P value | 95% CI | | OR | P value | 95% CI | | OR | P value | 95% CI | |
| Surgery (no) |  |  |  |  |  |  |  |  |  |  |  |  |
| Yes | 1.750 | 0.005 | 1.187 | 2.580 | 2.041 | <0.001 | 1.371 | 3.037 | 2.146 | <0.001 | 1.437 | 3.204 |
| Age (45-55 years) |  |  |  |  |  |  |  |  |  |  |  |  |
| 55-65 years | 0.820 | 0.383 | 0.525 | 1.281 | 1.446 | 0.123 | 0.905 | 2.310 | 1.318 | 0.245 | 0.828 | 2.097 |
| ≥65 years | 1.108 | 0.676 | 0.684 | 1.797 | 1.551 | 0.095 | 0.926 | 2.597 | 1.435 | 0.171 | 0.856 | 2.404 |
| Gender (male) |  |  |  |  |  |  |  |  |  |  |  |  |
| Female | 1.197 | 0.374 | 0.805 | 1.781 | 0.686 | 0.060 | 0.463 | 1.016 | 0.786 | 0.239 | 0.527 | 1.173 |
| Marital status (married/partnered) |  |  |  |  |  |  |  |  |  |  |  |  |
| Never married/divorced | 1.023 | 0.942 | 0.557 | 1.879 | 0.763 | 0.423 | 0.394 | 1.478 | 0.474 | 0.044 | 0.229 | 0.982 |
| Residence location (urban area) |  |  |  |  |  |  |  |  |  |  |  |  |
| Rural area | 1.702 | 0.006 | 1.162 | 2.493 | 0.923 | 0.685 | 0.628 | 1.358 | 1.637 | 0.015 | 1.100 | 2.436 |
| Region (east) |  |  |  |  |  |  |  |  |  |  |  |  |
| Central | 0.883 | 0.552 | 0.587 | 1.330 | 1.569 | 0.035 | 1.031 | 2.388 | 1.513 | 0.056 | 0.989 | 2.316 |
| West | 0.915 | 0.721 | 0.563 | 1.488 | 1.334 | 0.270 | 0.800 | 2.227 | 1.415 | 0.184 | 0.848 | 2.363 |
| Health insurance (no) |  |  |  |  |  |  |  |  |  |  |  |  |
| Yes | 1.049 | 0.885 | 0.549 | 2.005 | 1.189 | 0.614 | 0.607 | 2.328 | 1.000 | 1.000 | 0.511 | 1.957 |
| SES Index (low level) |  |  |  |  |  |  |  |  |  |  |  |  |
| High level | 1.062 | 0.753 | 0.730 | 1.545 | 1.543 | 0.027 | 1.051 | 2.264 | 0.913 | 0.651 | 0.617 | 1.353 |
| Constant | 0.233 | <0.001 | 0.104 | 0.520 | 0.175 | <0.001 | 0.076 | 0.404 | 0.178 | <0.001 | 0.077 | 0.412 |

Note: Logistic regressions adjusted for: age, gender, marital status, residence location, region, health insurance and socioeconomic level.

**Table S8** The impacts of the Radiation therapy on health service use and catastrophic health expenditure

| **Variables (reference)** | **Outpatient**  **visits** | | | | **Admission**  **to hospital** | | | | **Catastrophic health expenditure** | | | |
| --- | --- | --- | --- | --- | --- | --- | --- | --- | --- | --- | --- | --- |
|  | OR | P value | 95% CI | | OR | P value | 95% CI | | OR | P value | 95% CI | |
| Radiation therapy (no) |  |  |  |  |  |  |  |  |  |  |  |  |
| Yes | 0.845 | 0.660 | 0.400 | 1.788 | 3.310 | <0.001 | 1.714 | 6.393 | 1.675 | 0.138 | 0.847 | 3.314 |
| Age (45-55 years) |  |  |  |  |  |  |  |  |  |  |  |  |
| 55-65 years | 0.806 | 0.341 | 0.517 | 1.256 | 1.326 | 0.238 | 0.830 | 2.118 | 1.237 | 0.365 | 0.781 | 1.958 |
| ≥65 years | 1.040 | 0.873 | 0.645 | 1.676 | 1.423 | 0.177 | 0.853 | 2.375 | 1.295 | 0.319 | 0.779 | 2.151 |
| Gender (male) |  |  |  |  |  |  |  |  |  |  |  |  |
| Female | 1.174 | 0.428 | 0.790 | 1.746 | 0.729 | 0.116 | 0.492 | 1.081 | 0.804 | 0.282 | 0.540 | 1.196 |
| Marital status (married/partnered) |  |  |  |  |  |  |  |  |  |  |  |  |
| Never married/divorced | 1.031 | 0.922 | 0.563 | 1.889 | 0.772 | 0.442 | 0.398 | 1.495 | 0.486 | 0.051 | 0.236 | 1.002 |
| Residence location (urban area) |  |  |  |  |  |  |  |  |  |  |  |  |
| Rural area | 1.668 | 0.008 | 1.142 | 2.435 | 0.931 | 0.715 | 0.633 | 1.369 | 1.615 | 0.017 | 1.090 | 2.392 |
| Region (east) |  |  |  |  |  |  |  |  |  |  |  |  |
| Central | 0.861 | 0.469 | 0.574 | 1.292 | 1.558 | 0.039 | 1.023 | 2.372 | 1.461 | 0.077 | 0.960 | 2.223 |
| West | 0.871 | 0.574 | 0.537 | 1.411 | 1.329 | 0.276 | 0.797 | 2.216 | 1.346 | 0.250 | 0.811 | 2.233 |
| Health insurance (no) |  |  |  |  |  |  |  |  |  |  |  |  |
| Yes | 1.014 | 0.967 | 0.532 | 1.930 | 1.175 | 0.639 | 0.599 | 2.304 | 0.970 | 0.929 | 0.499 | 1.887 |
| SES Index (low level) |  |  |  |  |  |  |  |  |  |  |  |  |
| High level | 1.074 | 0.705 | 0.741 | 1.558 | 1.606 | 0.016 | 1.093 | 2.359 | 0.942 | 0.764 | 0.639 | 1.389 |
| Constant | 0.305 | 0.003 | 0.139 | 0.668 | 0.199 | <0.001 | 0.088 | 0.451 | 0.235 | <0.001 | 0.105 | 0.527 |

Note: Logistic regressions adjusted for: age, gender, marital status, residence location, region, health insurance and socioeconomic level.
